# Exhausted Heart Rate Responses to Repeated Psychological Stress in Women With Major Depressive Disorder

**DOI:** 10.3389/fpsyt.2022.869608

**Published:** 2022-04-18

**Authors:** Carmen Schiweck, Ali Gholamrezaei, Maxim Hellyn, Thomas Vaessen, Elske Vrieze, Stephan Claes

**Affiliations:** ^1^Department for Psychiatry, Psychosomatic Medicine and Psychotherapy, University Hospital, Goethe University, Frankfurt, Germany; ^2^Psychiatry Research Group, Department of Neurosciences, KU Leuven, Leuven, Belgium; ^3^Faculty of Medicine and Health, Pain Management Research Institute, The University of Sydney, Sydney, NSW, Australia; ^4^University Psychiatric Centre, KU Leuven, Leuven, Belgium

**Keywords:** stress reactivity, heart rate variability, allostatic load, depression, biomarker, repeated stress

## Abstract

Past research links depression and blunted cardiac vagal reactivity to chronic stress. Yet, to our knowledge no experiment investigates heart rate (variability) responses to a repeated laboratory stressor in patients with depression. Repeated exposure may provide valuable information on stress reactivity in depression. Fifty-nine women (30 inpatients diagnosed with depression and 29 matched controls) underwent two consecutive runs of a mental arithmetic stress paradigm consisting of one baseline and two exposures to control, stress, and recovery phases of 5 min each, in a case-control design. Subjective stress and electrocardiography were recorded. Variance of heart rate (HR) and root mean square of successive RR interval differences (RMSSD) were analyzed using linear mixed models. Overall, physiological parameters (HR and RMSSD) and subjective stress showed a strong group effect (all *p* < 0.001). In both groups, subjective stress and HR increased in response to stress, but the subjective stress levels of patients with depression did not return to baseline levels after the first stressor and for the remainder of the experiment (all *p* < 0.004 compared to baseline). Patients’ HR reactivity responded oppositely: while HR recovered after the first stress exposure, no reactivity was observed in response to the second exposure. These findings may suggest that the often-reported blunted HR/HRV response to stressors results from exhaustion rather than an incapacity to react to stress. The altered HR reactivity could indicate allostatic (over-) load in depression.

## Introduction

Major Depressive Disorder (MDD) is currently diagnosed in an estimated 300 million people worldwide (1), conferring profound disability for those affected and an incremental economic burden for society (2). That the experience of psychological stress is among the most critical factors preceding the development of a first depressive episode has long been established (3, 4) and it is now also clear that stress acts not only as a catalyst, but also as an important factor for the maintenance of depression (5, 6). This auto-conserving cycle of chronic stress exposure and depressive episodes does bear deep and long-lasting consequences for underlying psychological and biological systems (7). Next to a generally overactive hypothalamus-pituitary-adrenal (HPA) axis (8, 9) and altered immunological reactivity (10), the Autonomic Nervous System (ANS) also shows significantly altered activity in both patients with clinical depression (11–14) and healthy participants with depressive symptoms (15–17).

We recently reviewed the literature to investigate physiological reactivity to self-relevant and active stressors in patients with clinical MDD (18). The majority of studies showed an overall blunted response during stress or impaired recovery in MDD, both for heart rate (HR) and heart rate variability (HRV). Contemporary theories of depression pathogenesis posit that this blunted stress reactivity is the effect of allostatic overload (19–21). Allostatic overload can be defined as dysregulation or wear and tear of the body, reflecting a state of physiological exhaustion, brought about by chronic and recurrent exposure to stressors. The mechanisms leading to such exhaustion include the failure to return to homeostasis after the stressor is eliminated, an insufficient response to cope with a stressor and the failure to physiologically and/or psychologically adapt to repeated stressors [also see Guidi et al. (22) for a detailed overview of these postulates]. The first and second of these factors are relevant for both the clinical assessment of allostatic load (20, 23) and for the clinical/biological presentation and treatment of MDD.

Regarding adaptation to repeated stressors, in an interesting recent article, the group of Ginty has investigated the cardiovascular adaptation capacity to repeated stressors in healthy controls using an identical stressor within one session (24). The authors have shown that greater perceived life stress was associated with blunted HR responses and less adaptation to the repeated stressor. However, they did not account for depressive symptoms, nor did they assess a patient group. As a result, it is still unclear whether the cardiovascular blunting of the stress response in patients with depression is due to chronic stress itself rather than depression and if, or how, the magnitude of the acute response is modulated by repeated stress experience in patients with depression. The quick adaptability of HR/HRV responses may provide deeper insights in the acute mechanisms linked to allostatic overload. Understanding the exact nature of the presumed exhaustion (i.e., gradual blunting) may not only advance the understanding of stress-related disorders and the further development of the allostatic load model but can also open new treatment avenues. To clarify whether and how repeated exposure to a stressor is altered in depression, and how this is affected by chronic stress we here set out to explore the effect of an identical repeated stressor on HR and HRV in patients with depression in response to an active stress task.

Several factors need to be taken into consideration in order to assess the above questions. Firstly, patients with MDD commonly encounter recurrent stressors of the same nature (3). Yet, to our knowledge, all current laboratory studies used one single, or two different stressors when investigating stress reactivity in MDD, showing (mostly) blunted stress reactivity to active stressors and (mostly) hyperactivity to passive stressors [for an overview see Schwerdtfeger and Rosenkaimer (17)]. Therefore, for the first time, we here explore the effect of an identical, repeated stressor on cardiac parameters in patients with depression in response to an active stress task within the same session.

Secondly, it is well known that variables such as sex (25, 26), age (27), smoking status (28), antidepressant medication (29) and Body Mass Index (BMI) (30) influence HR/HRV and potentially physiological stress reactivity and should be taken into account to draw firm conclusions. To address these potentially confounding factors, we here opted to focus on women, and match patients with MDD and controls for age and where possible BMI, and restricted smoking immediately prior to the experiment. We chose women, since depression is more prevalent in women and it has been suggested that blunting of the stress response may be particularly relevant (31).

In addition, the effect of chronic stress is highly relevant for stress reactivity analyses in patients with depression, since stress/stressful life events often precede the onset of depressive symptoms (14, 32) and chronic stress *per se* may be linked to blunted stress reactivity: Ginty et al. (33) showed that in healthy individuals, higher perceived stress (compared to objective stress exposure) was linked to blunted cardiovascular stress reactivity (33). However, the effect of perceived stress is often not examined in patients with depression. To disentangle the effects of stress and depression diagnosis we will therefore attempt to assess the moderating role of chronic stress levels on the effect of depression diagnosis. Lastly, recent studies have successfully used HR/HRV as biomarker for MDD (34–36). In order to use HR reactivity during stress as stratification marker for new treatment options, this would require a reasonable sensitivity and specificity for depression. Therefore, we will here attempt to replicate stress reactivity as marker to distinguish patients with MDD.

Thus, we here explore HR and HRV reactivity to an identical, repeated stressor in a carefully controlled design to test whether (a) stress reactivity/recovery to a repeated stress task is altered in depression (exploratory analysis); (b) higher levels of (perceived) chronic stress are associated with a blunted stress response; (c) whether this is different in healthy individuals compared to patients; and lastly, (d) whether HR/HRV reactivity to- and recovery from stress can be used to distinguish patients and controls. We hypothesize that (a.1.) congruent with the literature, HR reactivity to the first stressor is blunted and (a.2.) that recovery from stress is impaired in patients with MDD compared to controls. Additionally, we explore whether the second stress exposure differs from the first exposure with regard to magnitude (e.g., increased blunting, increased reactivity, or no qualitative change); for (b) we hypothesize that participants with depression who have higher levels of chronic stress show blunted stress reactivity; (c) in contrast, we expect that healthy controls with high levels of chronic stress show increased reactivity; and lastly that (d) combined HR and root mean square of successive difference (RMSSD) reactivity during stress and/or recovery can be used to distinguish patients and controls.

## Materials and Methods

This study was performed according to Good Clinical Practice and approved by the local ethics committee (EC number: S59441, UZ Leuven Medical Ethics Committee, Belgium). Participants gave written informed consent prior to study related activities. The study recruitment and tests ran from 2016 to 2018.

### Study Design

**FIGURE 1:**
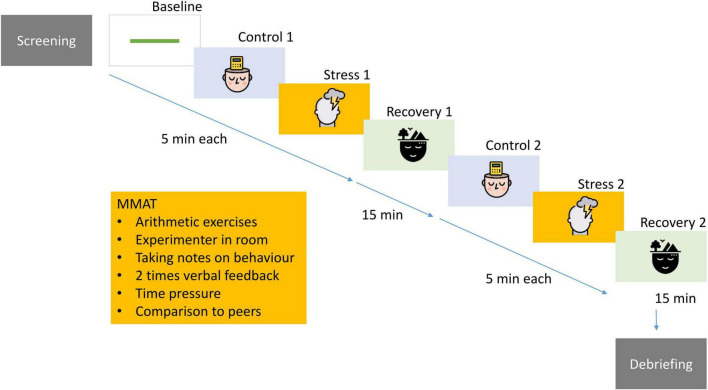
Participants underwent extensive psychological screening and completed a computerized, repeated version of the mental arithmetic stress task including social evaluative components. This procedure was administered twice in one session. For a complete description see Supplementary Information A. Design: “images: Flaticon.com.” This figure has been designed using resources from Flaticon.com. Stress icons created by Freepik – Flaticon (https://www.flaticon.com/free-icons/stress). Psychology icons created by iconixar – Flaticon (https://www.flaticon.com/free-icons/psychology). Calm icons created by Becris – Flaticon (https://www.flaticon.com/free-icons/calm).

The study consisted of a screening visit during which the diagnostic interviews and questionnaires were administered, and a test session held between 12.00 and 18.30 during which participants performed two runs of the 5 min repeated mental arithmetic stress task separated by 15 min of break. During the entire task, participants were wearing an ECG. See Figure 1 for an overview of the study design and below for detailed procedures.

#### Participant Selection and Procedure

Thirty women with MDD (56% undergoing antidepressant pharmacological therapy) and 29 women without mental health diagnosis matched for age (tolerance ± up to 5 years) and where possible BMI, underwent an extensive diagnostic interview (MINI PLUS Version 6.0.0, Dutch version) following the Diagnostic and Statistical Manual of Mental Disorder 4th edition (DSM–IV). Patients were currently treated for depression as in- or outpatients in the UPC KU Leuven Psychiatric Hospital. Controls had to be free of any lifetime psychiatric disorder. All participants had to be free of cardiovascular disease based on self-report and were excluded if they were taking medication with significant effects on cardiovascular function (e.g., antihistamines, beta-blockers, and tricyclic antidepressants) at the exception of SSRIS, SNRIS, atypical antidepressants or benzodiazepines (as on-demand medication), and contraceptive medication. Demographic data including menstrual cycle phase was recorded. One control participant and 3 patients had to be excluded from analysis [due to prohibited medication on the testing day (antihistamine) *n* = 1, arrhythmias *n* = 1 discovered during analysis of data, drop-out before stress task *n* = 2], leaving a total sample of 28 controls and 27 patients.

#### Questionnaires

The 10-item version of the self-rated Perceived Stress Scale (PSS) was used to assess levels of chronic stress (37). It is a well-validated scale commonly used to assess stress perception during the past 4 weeks and has good psychometric properties (38). The Dutch self-rated version of the Rumination Response scale (RRS) was used to assess whether patients had higher levels of rumination than controls. The Dutch RRS has shown good reliability and satisfactory validity (39). The General Practice Physical Activity Questionnaire was used to assess activity levels of participants yielding four different categories of physical activity from inactive to very active [National Collaborating Centre for Nursing and Supportive Care (United Kingdom) (40)]. The self-rated, Depression Anxiety and Stress Scale (DASS) 21-item version was used to assess general symptoms of stress, depression and anxiety in the past week (41, 42). It has good internal consistency and validity was also good in Dutch participants (43). In addition, to assess depressive symptoms, the Hamilton Rating Scale for Depression (HRSD) 17-item version was used to assess depression severity in participants (44, 45). The HRSD is the gold standard to assess clinician-rated, clinical symptoms of depression and has generally good validity and reliability (46).

#### Repeated Modified Mental Arithmetic Task

Detailed information on the study design can be found in Supplementary Information 1. In short, the repeated modified mental arithmetic task (RMMAT) was computed in Affect version 4.0 (47). It is a modified version of the mental arithmetic/Montreal Stress Imaging task, and includes a baseline, a control task (i.e., a mental arithmetic without stressful elements), a mental arithmetic (stress), and a recovery phase. In this modified, computerized version, several elements for social evaluation, time pressure and feedback by the researcher are integrated to induce stress during the stress phases. After the first exposure to the RMMAT and the first recovery period, the researcher asked the participants unexpectedly to perform the RMMAT a second time. The participants were not aware in advance that this second administration would take place. This was done on purpose to be able to assess the reactivity to repeated stressors without confounding the effect by anticipatory stress.

#### Procedure

Within 7 days after the screening visit (including the diagnostic interview and questionnaires), participants underwent the RMMAT. If more than 7 days had elapsed between screening and the stress task, questionnaires were re-administered. To avoid bias, participants were instructed not to smoke at least 1 h prior to the start of the experiment, were asked not to consume caffeine and restrain from sport on the day of, and from heavy exercise the day prior to the experiment.

Upon arrival, participants were placed in a comfortable chair in front of a computer, instructed to find a comfortable position and move as little as feasible. Electrodes for cardiovascular measurement and a respiratory belt were attached as described below and tested for proper functioning. Next, the experimenter explained the setup of the experiment, omitting to mention the second exposure to the stress task. After the first stress task the experimenter re-entered the room and informed participants that unexpectedly, they had to perform the task again. At baseline, after the stress and during the recovery phases of both stress exposure experiments, participants were asked to rate their stress level on a 10-point Likert scale (0 = not at all to 10 = extremely stressed). Total task duration was approximately 60 min and the time of assessment was between 12.00 and 18.30 (i.e., after the diurnal normal increase in HR and before the diurnal drop in the evening). The choice to use the timeslot between 12.00 and 18.30 was based on our pilot study on diurnal variations in HR in patients with MDD and healthy controls, where a prominent increase in basal HR occurred during the morning, whereas the most significant decrease occurred later in the evening. The period-between 12.00 and 18.30 yielded a plateau ideal for behavioral analyses (48). Participants were in a seated position in a room with a controlled temperature of 22°C. After completion, participants were debriefed, thanked and discharged.

### Electrocardiography and Respiratory Recording

Cardiac electrical activity (ECG) and respiratory movement were recorded using a 10-channel isolated bioamplifier (NeXus-10 MKII, Mind Media BV, Netherlands). Three disposable electrodes (Kendall™, H66LG electrodes) were placed in a lead II configuration on the chest to record ECG at sampling frequency of 1,024 Hz. Simultaneously, respiratory (chest) movement was recorded by placing a strain gauge belt over the abdomen, with the central part just above the navel and a sampling rate of 32 Hz was used, as instructed by the manufacturer. Correct functioning of the recordings was checked using the BioTrace+ Software (Mind Media BV, Netherlands).

### Data Analysis and Cleaning

The ECG signal was processed and R-wave peaks were detected using Kubios software version 2.0 (49). A visual inspection of raw data for possible movement artifacts and ectopic beats was performed per segment (i.e., each 5-min interval). Where applicable, an inbuilt artifact correction based on cubic spline interpolation was applied. Segments with more than 5% of beats in need for correction were excluded from analysis (one segment). Data was excluded for one patient showing a high number of ectopic beats (arrhythmia), and two segments of data were lost for two participants due to equipment failure. The respiratory movement signal was processed using custom-written algorithms in MATLAB software (R2018b, Mathworks, Inc., Natick, MA, United States), see Gholamrezaei et al. (50), and breathing rate was measured for each segment.

### Heart Rate and Heart Rate Variability

Heart rate variability analysis was performed using the Kubios software on segments with duration of 5 min according to the guidelines (51). HR, RMSSD, and high-frequency HRV (HF-HRV) were extracted for each segment. RMSSD is a time-domain HRV index reflecting beat-to-beat variation in HR, and HF-HRV is a frequency-domain HRV index reflecting variability of HR at spontaneous breathing frequency (i.e., respiratory sinus arrhythmia or RSA). Both indexes are mainly derived by cardiac parasympathetic (vagal) activity as is shown by studies using vagolytic (e.g., high dose atropine) (52–55) or vagotonic (e.g., low dose atropine) agents (56–58) to inhibit or increase cardiac parasympathetic stimulation, respectively (59). We decided to use RMSSD as the primary HRV index in our analysis for specific reasons: Evidence has shown that RMSSD is less influenced by breathing behavior, particularly the breathing frequency, compared to HF-HRV (54, 55, 60). Controlled breathing is recommended for a reliable assessment of cardiac vagal activity when using frequency-domain measures (54, 55). Therefore, RMSSD is a more suitable measure of cardiac vagal activity when controlled breathing is not possible/feasible, as was the case in the current study. Notwithstanding, to provide more comparable data with previous studies, we decided to also analyze HF-HRV while taking breathing frequency into account. Results of HF-HRV and for completeness, low frequency (LF-HRV) are provided in the Supplementary Information D.

### Statistical Analysis

Statistical analyses were performed in R (61) and matching of participants and controls was performed using SPSS version 25 (62). The power analysis on simulated data can be found in Supplementary Information B. We conducted a repeated-measures mixed model with random intercept, a phase (baseline, stress, recovery) × group (MDD/control) interaction and PSS × group interaction as independent variables, and either subjective stress rating, or HR/HRV as dependent variables. Assumptions of mixed models were checked by visual inspection of q–q-plots and histograms. Due to slight heterogeneity of residuals, HR and HRV indexes were transformed using the logarithmic (log) transformation for all statistical analyses. The initially planned interaction of PSS × phase to test the moderating effect was collinear with the group × phase interaction and was therefore not explored in the overall model. Nevertheless, to investigate this effect we conducted a separate model within the groups (either MDD or HC) with a PSS × phase. Significant time × group interactions were followed-up by planned contrasts adjusted for multiple testing using Benjamini–Hochberg correction between baseline and stress, between stress and recovery and between baseline and recovery of each run. Since no difference occurred between the baseline and the control condition (see Supplementary Information C), and the baseline phase was more comparable to the recovery phases, the control condition was not considered in the model.

To investigate classification accuracy of physiological parameters, we used binary logistic regression with leave one out cross validation (LOOCV) from the caret package (50).

## Results

### Demographic Data and Baseline Differences

Patients and controls were female, between 18 and 60 years old, matched for age (using fuzzy matching, tolerance up to ±5 years) with a similar BMI. Menstrual phases (contraception, post-menopausal, follicular, or luteal phase) did not differ significantly between groups (*X*^2^ = 3.54, df = 3, *p* = 0.315). The mean HRSD score for patients was 24.63 indicating a severe form of depression (51). Controls were more likely to be non-smokers (*X*^2^ = 13.55, df = 2, *p* = 0.001) and were significantly more active than patients (*W* = 634, *p* < 0.001). Additionally, patients had significantly higher depression severity, rumination- and perceived stress levels in the last month (all *p* < 0.001, see Table 1). None of the control population and around half (15 of 27) of the patients were taking SSRIS/SNRIs/SARIS (*n* = 11) or atypical antidepressants (*n* = 4). Four patients were exclusively treated with benzodiazepines.

**TABLE 1:** Demographic data and statistical comparison.

Variable of interest	HC	*n* = 28	MDD	*n* = 27	Test statistic		
	Mean	SD	Mean	SD	*X*^2^/*t*	*p*	sig
Age	32.07	10.62	33.22	12.47	−0.37	0.715	ns
BMI	23.33	3.9	25.18	5.32	1.47	0.149	ns
Contraception, *n* (%)	17	(61%)	11	(41%)	2.16	0.142	ns
Post-menopausal, *n* (%)	4	(14%)	4	(15%)	0.011	0.917	ns
Luteal phase, *n* (%)	3	(11%)	8	(30%)	3.01	0.083	ns
Follicular phase, *n* (%)	4	(14%)	4	(15%)	0.011	0.917	ns
Smoker, current, *n* (%)	1	(4%)	11	(41%)	10.71	0.001	**
Non-smoker, *n* (%)	25	(89%)	12	(44%)	12.34	<0.001	***
Smoker, past, *n* (%)	2	(7%)	4	(15%)	0.89	0.346	ns
Physical activity	3.86	0.67	2.37	1.10	634#	<0.001	***
Depression severity (HRSD)	1.71	1.88	24.63	5.66	0#	<0.001	***
Perceived Stress Score (PSS)	8.86	3.94	28.56	6.45	18#	<0.001	***
Rumination Response Scale (RRS)	8.54	8.14	48.56	12.05	2#	<0.001	***
Antidepressant medication, *n* (%)	0	0%	15	56%	–	–	

ns, not significant; ** significant at p < 0.01; *** significant at p < 0.001; # Wilcoxon rank sum test statistic.

### Effect of Covariates on Baseline Levels of Heart Rate/Root Mean Square of Successive Difference

While antidepressant medication intake was limited to SSRIs/SNRIs/SARIs and atypical antidepressants and smoking immediately prior to the experiment (at least for 1 h) was prohibited, these parameters may constitute an important confounder. We therefore tested whether the demographic variables that were different between controls and patients (i.e., medication, smoking, and physical activity levels) contributed to HR/HRV group differences at baseline. No main or moderating effect occurred for smoking (all *p* > 0.231) or physical activity (all *p* > 0.765). Antidepressant medication had no main effect on HR at baseline (*t* = −0.33, *p* = 0.748), but significantly decreased RMSSD (*t* = −2.46, *p* = 0.021).

### Subjective Stress in Response to the Stress Task

**FIGURE 2:**
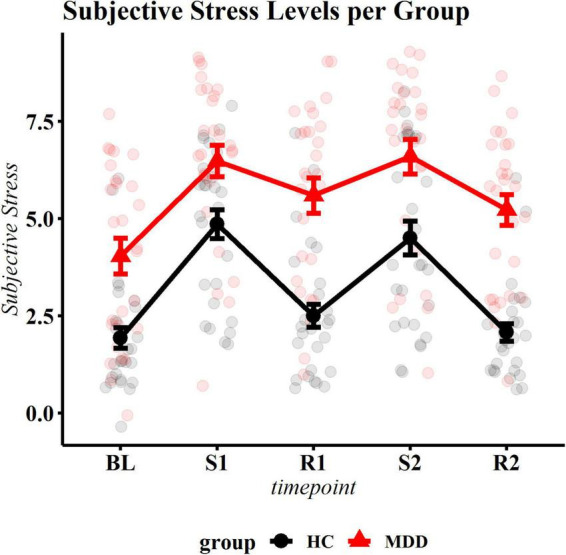
Subjective stress ratings per phase. The black (bottom) line represents control participants, the red (top) line represents patients. BL, baseline; S1, first stressor; R1, first recovery phase; S2, second stressor; R2, second recovery phase. Summary statistics indicate mean ± standard errors. All change scores were significantly different for the control group. Patients and healthy control individuals showed significant reactivity to the first stressor. In healthy individuals, stress levels recovered after both stress exposures, while patients did not return to baseline.

As a manipulation check, we tested whether subjective stress induction was achieved. A significant group by phase interaction occurred (*F* = 3.11, df = 4, *p* = 0.016). *Post hoc* contrasts of phase by group showed the expected increase of SS in healthy controls, during both runs (compared to baseline *t* = 7.57, *p*.adj < 0.001 and *t* = 6.64, *p*.adj < 0.001), returning to approximate baseline levels during recovery (*t* = −1.48, *p*.adj = 0.154 and *t* = −0.37, *p*.adj = 0.712). Congruent with the control’s stress response, in patients, SS increased in response to the first stressor (*t* = 6.21, *p* < 0.001). However, while there was a subtle recovery after stress (*t* = −0.89, *p*.adj = 0.030), SS levels did not return to baseline during the recovery period (*t* = −3.95, *p* < 0.001) and stayed significantly elevated throughout the remainder of the experiment (all *p* < 0.004 compared to baseline), indicating incomplete subjective stress recovery (Figure 2 and Supplementary Table 1).

### Heart Rate Reactivity to Stress

**FIGURE 3:**
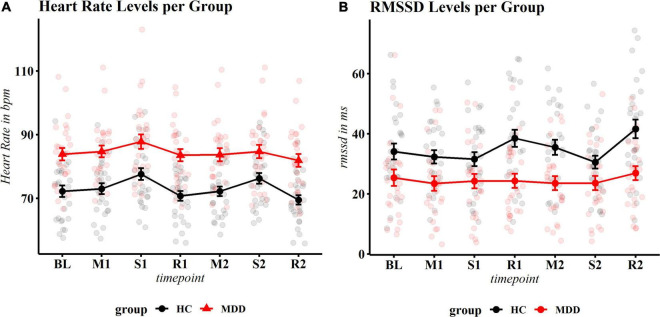
Physiological reactivity to stress. **(A)** Heart rate (HR): significant overall reactivity and a group × timepoint interaction occurred. Controls show significant reactivity and recovery, while patients show an increase of HR to the first stressor and a decrease during recovery 1, but no longer react to the second stressor (neither compared to BL nor compared to recovery 1). **(B)** Root mean square of successive differences (RMSSD). Controls show significant response after the first stressor (recovery 1), the second stressor and second recovery, MDD patients show no fluctuation at any time point. Heart rate is displayed in beats per minute; MDD, Major Depressive Disorder; HC, healthy controls.

Mean levels of HR and RMSSD reactivity can be found in Supplementary Table 2. The final model included a group × phase interaction (and respective main effects), a group × PSS interaction (and respective main effects) and antidepressant medication. The PSS × group interaction (*F* = 10.41, *p* = 0.002) and the group × phase interaction (*F* = 2.99, *p* = 0.019), and antidepressant medication (*F* = 5.78, *p* = 0.020) were significant. Patients with MDD had overall higher HR throughout the experiment (Figure 3A). *Post hoc* contrasts revealed that in controls, HR increased significantly during the first (*t* = −5.11, *p*.adj < 0.001) and second stressor compared to baseline (*t* = −4.01, *p*.adj = 0.001) and returned to approximate baseline levels (or slightly lower levels) during first (*t* = 0.89, *p*.adj = 0.421) and second recovery phase (*t* = 2.02, *p*.adj = 0.080) compared to baseline (see Table 2 for exact statistics).

**TABLE 2:** Results of planned within group contrasts for split linear mixed models for HR reactivity.

Controls									Patients with MDD					
Contrast			Estimate	SE	df	*t* ratio	*p*-Value	*p*.adj	Estimate	SE	df	*t* ratio	*p*-Value	*p*.adj
BL	–	S1	−0.077	0.015	221.5	−5.105	**<0.0001**	**<0.0001**	−0.045	0.015	221.3	−2.901	**0.004**	**0.011**
BL	–	R1	0.013	0.015	221.5	0.890	0.375	0.421	0.007	0.016	221.5	0.431	0.667	0.667
BL	–	S2	−0.060	0.015	221.5	−4.011	**<0.001**	**<0.001**	−0.010	0.015	221.3	−0.668	0.505	0.534
BL	–	R2	0.030	0.015	221.5	2.022	0.044	0.080	0.024	0.016	221.4	1.509	0.133	0.217
S1	–	R1	0.090	0.015	221.3	6.063	**<0.0001**	**<0.001**	0.051	0.016	221.5	3.297	**0.001**	**0.003**
S1	–	S2	0.016	0.015	221.3	1.106	0.270	0.346	0.034	0.015	221.3	2.233	**0.027**	0.060
R1	–	S2	−0.074	0.015	221.3	−4.956	**<0.0001**	**<0.001**	−0.017	0.016	221.5	−1.091	0.276	0.346
R1	–	R2	0.017	0.015	221.3	1.145	0.253	0.346	0.017	0.016	221.6	1.065	0.288	0.346
S2	–	R2	0.091	0.015	221.3	6.101	**<0.0001**	**<0.001**	0.034	0.016	221.4	2.169	**0.031**	0.062

*Controls (left) and patients (right) with MDD. Significant results are printed in bold. BL, baseline; S1, stressor 1; R1, recovery phase 1; R2, recovery phase 2; SE, standard error; p.adj, p-values adjusted for multiple comparisons with BH correction and for use of antidepressant medication (0 = no/1 = yes).*

In patients with MDD, HR significantly increased during the first stressor (*t* = −2.90, *p*.adj = 0.011 compared to baseline) and returned to baseline levels during recovery (*t* = 0.43, *p*.adj = 0.667). However, no significant reaction to the second stressor was observed, neither compared to baseline, nor the first recovery period (*t* = −0.67, *p*.adj = 0.534 and *t* = −1.09, *p*.adj = 0.346, respectively). During the second recovery phase, a non-significant reduction of HR occurred compared to the second stressor (*t* = 2.16, *p*.adj = 0.062) (Figure 3A). Exploration of the significant group × PSS interaction showed an inverse U shape at baseline, where high stress levels were associated with higher HR in healthy controls, but with lower HR in patients with depression (see Supplementary Figure 1). For an overview of planned contrasts per phase for patients and controls, see Table 2. Of note, in an additional exploratory model within the MDD group, the main effect of PSS (*F* = 19.16, *p* = 0.002), but not the phase × PSS interaction (*F* = 1.22, *p* = 0.306) was significant, indicating that chronic stress influenced overall HR levels but did not (additionally) alter the reactivity in patients with MDD. No effect of PSS (*F* = 1.26, *p* = 0.271) or the phase × PSS interaction (*F* = 0.370, *p* = 0.830) occurred for healthy controls. It should however be noted that healthy controls had in general much lower stress levels than patients with MDD and an exact disentanglement of group and stress effects was therefore not possible.

### Root Mean Square of Successive Difference Reactivity to Stress

For RMSSD reactivity, group (*F* = 13.98, *p* < 0.001), phase (*F* = 6.36, *p* < 0.001) and a group × PSS interaction (*F* = 4.26, *p* = 0.043) were significant. Antidepressant medication use was not a significant predictor (*F* = 0.35, *p* = 0.557), but since RMSSD is known to be influenced by this measure, was kept in the model. *Post hoc* contrasts by group revealed that controls showed significantly lower RMSSD levels during the second stress task when compared to the first recovery phase (*t* = 3.18, *p*.adj = 0.010) and significant recovery after the second stress exposure (*t* = −4.81, *p* < 0.001). Reactivity of the first stressor compared to baseline was not significant (*t* = −1.92, *p*.adj = 0.168). Patients with depression showed no significant reactivity at any time point (all *p*.adj > 0.170). The data is displayed in Figure 3B and Supplementary Table 2.

### Heart Rate During Recovery as Diagnostic Marker for Depression

**FIGURE 4:**
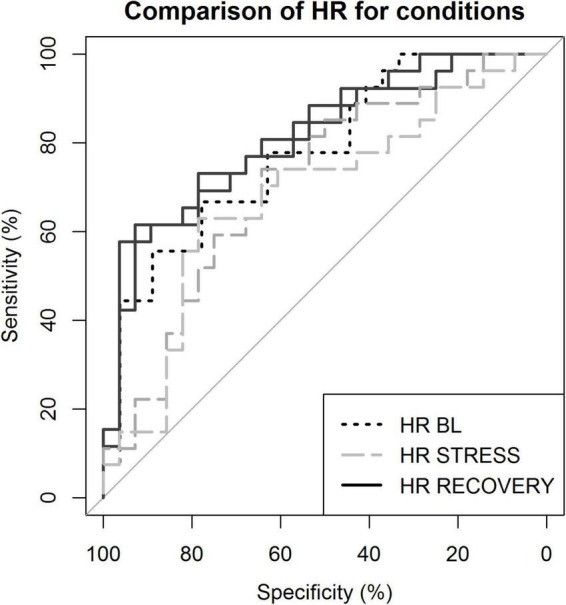
Receiver operating characteristic curves demonstrating sensitivity and specificity of the classification. Classification based on heart rate (HR) during various conditions. BL, baseline; STRESS, stress 1 and 2; and RECOVERY, recovery 1 and 2.

To assess if, and which phase of HR reactivity could best be used to distinguish depressed patients from controls, we performed binary logistic regression with LOOCV. The best distinction of patients and controls was achieved using HR with RMSSD during recovery. Supplementary Table 3 shows that, as a unique predictor, HR outperformed HRV indexes for classification. During recovery, combining these indexes led to a slightly better classification than for HR alone. Predictive accuracy was lowest during stress (65.45 and 63.63%) and highest during recovery from stress (first recovery 75.47% and second recovery 72.22%, respectively). Receiver operating characteristic (ROC) curves comparing various conditions can be found in Figure 4.

## Discussion

The present study aimed to explore the effect of repeated exposure to an identical stressor in depression. Results showed marked elevations in HR, lower vagally mediated HRV at rest and insufficient subjective stress recovery in women with depression. The results confirm previous findings showing blunted cardiac vagal modulation during stress in patients with MDD, e.g., Schiweck et al. (18) and Duschek et al. (63). In addition, we extend the literature by showing that repeated stress exposure provoked strongly reduced HR responses and poor subjective recovery in patients with depression, as opposed to healthy controls. Lastly, this pattern of cardiac reactivity differentiated between individuals with MDD and healthy volunteers with reasonably good accuracy and may have value as a complementary assessment tool for depression.

### Blunted Heart Rate Responses in Major Depressive Disorder: Adaptive Habituation or System Exhaustion?

As expected from the literature (64–66), our data clearly show overall higher subjective stress ratings in patients with MDD compared with healthy controls, both over the past month (as measured by the PSS) and during all phases of the experimental manipulation (as measured with the VAS). We here also replicate the finding that patients with depression show higher basal levels of HR and lower levels of HRV than controls (29), thereby confirming the representability of our sample. Most intriguing, however, is the finding that patients could not subjectively recover from stress and showed blunted reactivity to the second stressor.

Physiological habituation, conceived as significantly attenuated responses to a second stress exposure, can be influenced by many factors. In their review, Hughes et al. (67) conclude that healthy controls often show habituation of HR to stressors comparable to the here used MAT, but that this process can be disrupted by the presence and observation of the participants by the experimenter (60, 61, 68, 69). Since we designed the task to contain psychosocial stress elements including observation by the experimenter, it is not surprising that healthy controls do not show (significant) adaptation to the stressor. In our opinion, the psychosocial stress elements are crucial to test relevant stressors since psychosocial stress is highly important for depression pathogenesis. Opposed to subjective stress reactivity, HR levels in patients with depression did fully recover from the first stress exposure but did not show any reactivity to the second stressor. This may suggest a rapid habituation in MDD patients, despite the above-mentioned psychosocial stress elements, or alternatively may point to a dysfunctional or exhausted stress response. The first scenario would suggest that the observed physiological non-responsivity is an adaptive strategy to reduce energy expenditure. From this perspective, while essential to mount sufficient stress responses in the face of acute danger, a fast adaptation to a similar stressor would be very beneficial for energy resources. Yet, adaptive habituation would fail to account for the chronic high stress levels, increased HR and consistently blunted HR variability that have been observed in MDD (18, 70) and is therefore an unlikely hypothesis.

The physiological response could thus perhaps find a more integrative explanation when conceived as the consequence of allostatic load, as introduced by McEwen and Stellar (19, 21, 22, 71, 72). The authors propose that an initially adaptive mechanism to mobilize energy resources (allostasis) can lead to considerable wear and tear if it persists long term, as is the case with chronic stress (allostatic load). In line with these observations is a recent study: In a large population of healthy young adults, high levels of stress also led to blunted HR responses and additionally led to lower habituation over time (24), although the authors did not account for depressive symptomatology. In our sample, the group of healthy people was too small to investigate this differential effect in detail. However, we did observe a qualitatively different effect of chronic stress on baseline HR in healthy controls compared to patients with depression: While chronic stress increased HR in the controls, it decreased HR in patients with depression. It is therefore essential to account for this relationship in studies involving patients with MDD and would be highly informative to assess the effect of depressive symptoms in a large healthy working population.

The here observed discrepancy of subjective and physiological responses to stress in depression has also been found by others. In their study, Söder et al. found a higher discrepancy of recorded HR and reported subjective stress in patients with depression compared to controls during a noise stressor (73). This discrepancy of higher self-reported stress and relatively lower HR reactivity may contribute to the maintenance of chronic stress levels and deserves further investigation.

The repeated stress paradigm as tested in this study, albeit an artificial exposure, still seems highly relevant for the daily experience of patients with clinical depression, who often report a continuous feeling of high subjective stress and suffer from repeated minor stressors with which they seem unable to cope adequately. This inability to cope with repeated stressors might be somehow related to the decoupling of the biological stress response, caused by exhaustion. A next step to test this hypothesis would be to test patients with depression in daily life, carefully recording stressors, subjective responses, and physiological parameters using wearable devices.

### Blunted Cardiac Vagal Modulation to Psychosocial Stress

In response to the first stressor, patients with MDD showed increased subjective stress levels of approximately equal magnitude as controls, and a corresponding increase in HR. However, RMSSD showed nearly no variation throughout all phases of the task within the MDD population. While healthy controls also did not show vagal withdrawal to the first stressor, this observation could possibly be attributed to anticipatory stress levels at baseline. This is supported by the apparent recovery after the first stressor and significant reactivity to and recovery from the second stressor.

Ample research has documented blunted HR and HRV responses to stress in both participants with depressive symptoms (70) and patients with clinical depression (18). While we did not observe altered HR reactivity in patients with depression in response to the first stressor, the response to the second stress exposure was blunted, although the blunting could perhaps better be described as exhausted stress response (see section “Blunted Heart Rate Responses in Major Depressive Disorder: Adaptive Habituation or System Exhaustion?”). On the other hand, the association between depression and blunted cardiac vagal reactivity was confirmed and may point to consistently exhausted cardiac vagal modulation.

Blunted reactivity to stress in depression is not a novel concept in stress research: several reviews have investigated the matter with a focus on HPA axis reactivity. The most recent meta-analysis reveals that active, self-relevant stressors provoke similar, though sex-specific changes in cortisol release: whereas female participants with MDD show blunted reactivity to stress, male participants with MDD show increased responses (74). This corroborates findings with regard to the here described blunted cardiac vagal modulation in response to stress in our sample of female participants with MDD and highlights the importance of a careful study design accounting for relevant variables such as sex. The biological background behind this blunted reactivity alongside elevated HR to the first stressor may point toward primarily sympathetic activation rather than parasympathetic withdrawal during stress. This could be highly relevant for current therapies such as vagus nerve stimulation for depression, since restoring the balance between parasympathetic and sympathetic nervous systems should evaluate both sympathetic and parasympathetic output to quantify efficacy. However, as nerve activity was not assessed directly in this study, it is impossible to draw conclusions on this hypothesis.

### Decoupled Subjective and Physiological Stress Recovery: A Hallmark of Depression?

Despite the lack of (complete) subjective recovery after the first stress exposure, the patient group did show significant physiological recovery. This is interesting, as these results may inform about a possible process contributing to the maintenance of subjective stress levels: a failure to subjectively recover from minor stressors, as observed here, may lead to continuous maintenance of perceived stress and ultimately, increase the impact estimation of a stressor (66). This altered stress perception may also be the cause for the change in physiological parameters at baseline: e.g., chronic stress may increase basal HR levels and reduce parasympathetic activity.

The “*why’s*” and “*how’s*” of these physiological changes and their link to subjective stress can find a possible explanation in the literature of “*perseverative cognition*”: Brosschot et al. proposed that particularly rumination (both retrospective and prospective), as frequently encountered in depression, can negatively impact the consequences of psychological stress (75). As such, repetitive or “perseverative” thoughts about stress, and not necessarily the stressor itself, would lead to persistent physiological changes such as a chronically increased HR, blood pressure and altered cortisol levels (69). While the present study was not designed to directly test hypotheses related to perseverative cognition, it is conceivable that it impacts these physiological parameters. Particularly patients with depression did have high rumination scores. Yet, in this study, perceived stress rather than rumination scores increased model performance for the group with depression considerably, while rumination and perceived stress did not improve model fit for controls (data not shown). Therefore, preservative cognition may not be the most relevant factor for physiological changes in depression when accounting for chronic stress levels.

### Classification of Patients and Clinical Relevance: A Marker for Depression?

In the present study, the most accurate classification of patients could be achieved based on HR, with slightly improved classification when combined with RMSSD. Differences were largest during recovery from stress and lower accuracy was achieved during stress. Others have recently published results on classifying individuals with depression based on cardiovascular parameters, with similar accuracy as here reported. To illustrate, Kuang et al. found high classification accuracy (86.4%) with RMSSD throughout a series of physiological ANS stimuli (36). In a stress task, Byun et al. also found the clearest distinction with a HRV feature (SDNN) during stress recovery (34). Of note, although HR or HF-HRV were not the most important features reported by Byun et al., we here specifically focused on these parameters, given their biological relevance and relative robustness regarding interpretability.

The usefulness of an objective biomarker is obvious in a time where remote monitoring for the clinic becomes tangible. While this additional data does not replace diagnoses or contact with a clinician it may help making informed decisions for possible treatment augmentation, in patients with partial response to therapy, or for secondary prevention in recovered patients. These findings could also be particularly interesting in the light of cardiovascular health. This is certainly important in light of the increased risk for cardiovascular disease observed in depression (76). While we here did not have longitudinal data to ascertain whether HR could be used as a state marker, others have shown that in remitted patients, cardiovascular parameters normalized toward the control group (77). More recently, Hartmann et al. showed that in their brief longitudinal study, indeed, HF-HRV (significantly) and RMSSD (non-significantly) increased in patients with depression in the course of a 2-week antidepressant treatment regimen, emphasizing the need for further longitudinal studies to assess the feasibility of cardiovascular measurements as state markers for depression (78).

### Limitations

Firstly, the generalizability of this study is limited to female participants. Due to the well-documented sex differences in autonomic activity and sample sizes necessary to control for this effect, this was a necessary choice. However, menstrual status is known to have important effects on autonomic (re)activity. While recorded in this study, and an approximately equal proportion of patients and controls in each menstrual phase, analyses were underpowered to include this factor as covariate. Larger studies should include menstrual cycle as covariate and explore whether our findings are also applicable to men. It should also be mentioned that subjective stress experience and physiological reactivity were not always congruent. This well-known apparent incongruence (79) can arise from confounding factors such as age (80), sex (81) or the nature of the stressor. Other, external factors in larger samples may show different results. For instance, it has been shown that early life adversity can have profound effects on (physiological) stress reactivity (82), and future studies should account for this variable. In addition, habituation might better be tested in a stress paradigm without social stressors. However, this was not yet clear when this study was conceived in 2015, and social stress may actually be crucial to test ecologically relevant habituation (i.e., social compared to non-social stressors). A frequently reported measure for HRV analyses is LF-HRV. LF-HRV has previously been interpreted as reflecting cardiac sympathetic innervation. Yet, in resting conditions (as is the case here for parts of our experiments, the LF band reflects baroreflex activity and not cardiac sympathetic innervation (83). Interpretation of LF-HRV thus is complex and is not suitable to investigate our study aims. However, for the sake of completeness, it is reported in Supplementary Section. Finally, this is a cross-sectional study and therefore by definition, our results are correlational. Large-scale, longitudinal studies are needed to further investigate the potential of HR and HRV during stress as state markers for depression and its usefulness for the clinic.

### Conclusion and Future Research

We here show that stress reactivity is functional in patients with depression but appears exhausted in face of recurrent stress. The specific design of this study enabled us, for the first time, to experimentally demonstrate physiological (HR/HRV) exhaustion of the stress response in depression, lending support to Mc Ewen’s conception of “allostatic load” as destructive consequence of chronic stress, and in particular its importance for depression. Importantly, our results also show that re-exposure to a stressor of the same nature can be used to study exhaustion. Future studies should therefore replicate these findings and extend them to male subjects.

## Data Availability Statement

The raw data supporting the conclusions of this article will be made available by the authors, without undue reservation, doi: 10.17632/6jzfpzmks6.1.

## Ethics Statement

The studies involving human participants were reviewed and approved by the Ethische Commissie Onderzoek UZ/KU Leuven (EC Onderzoek). The patients/participants provided their written informed consent to participate in this study.

## Author Contributions

CS: conceptualization, data curation, formal analysis, investigation, methodology, project administration, software, visualization, writing—original draft, and writing—review and editing. AG: formal analysis, methodology, software, and writing-review and editing. MH: data curation, investigation, and writing—review and editing. TV: methodology, and writing—review and editing. EV: conceptualization, methodology, project administration, funding acquisition, supervision, roles/writing—original draft, and writing—review and editing. SC: conceptualization, funding acquisition, methodology, project administration, resources, validation, roles/writing—original draft, and writing—review and editing. All authors contributed to the article and approved the submitted version.

## Conflict of Interest

CS is a senior clinical researcher from the Fund for Scientific Research Flanders (FWO Vlaanderen). The remaining authors declare that the research was conducted in the absence of any commercial or financial relationships that could be construed as a potential conflict of interest.

## Publisher’s Note

All claims expressed in this article are solely those of the authors and do not necessarily represent those of their affiliated organizations, or those of the publisher, the editors and the reviewers. Any product that may be evaluated in this article, or claim that may be made by its manufacturer, is not guaranteed or endorsed by the publisher.
