# Supplementary material for: Exhausted Heart Rate Responses to Repeated Psychological Stress in Women With Major Depressive Disorder
Source: Front Psychiatry. 2022 Apr 18;13:869608. doi: 10.3389/fpsyt.2022.869608 (PMC9058080; doi:10.3389/fpsyt.2022.869608)
Supplement: Supplementary file 1 [file Data_Sheet_1.docx]

Supplementary Material

**Supplementary Information A**

**Repeated Modified mental stress task (RMMAT)**

We developed the RMMAT in Affect version 4.0 (Spruyt, Clarysse, Vansteenwegen, Baeyens, & Hermans, 2009) based on the well-validated mental arithmetic stress task and Montreal stress imaging tasks. The RMMAT is a computerized version of the mental arithmetic stress task which does not require verbal feedback of participants as this can infer with respiration. The RMMAT consists of 7 phases: a baseline phase, a control condition phase and a stress phase of 5 minutes each, followed by a recovery phase of 15 minutes (for HR/HRV analysis only the first 5 minutes of recovery were considered). Importantly, the subject does not know that the stress task will be repeated after the first exposure, to avoid apprehensive stress. During the baseline phase, the experimenter leaves the room and instructs the subject to RECOV. Journals with nature reports are provided for reading if desired. After the baseline phase, the experimenter re-enters the room and instructs the subject and answers any questions before starting the control condition task and leaving the room. The control condition task consists of several easy arithmetic tasks that the subject needs to solve by selecting the correct answer among three possibilities. There is no time pressure, and the subject does not receive any direct feedback from the experimenter.

The experimenter then re-enters the room and instructs the subject about the conditions of the stress task. The subject is told that she will play against other candidates with the same diagnosis age and academic background that were previously recorded. A bar at the side of the screen indicates the points of the other supposed participant which answers correctly in 90% of the questions. The researcher emphasizes how important it is that the participants perform well or at least as good as the other person so that he/she can use the results. The stress task consists of similar easy tasks as during the control condition task. However, every time the subject answers correctly, she will receive less time to answer the next question, making it impossible to keep answering at a given speed. In case the subject answers wrong, she will receive more time to answer the next question, such that the level of speed is automatically scaled by performance. The programme gives negative feedback to the subject “too slow” (visually)and “wrong” or “right” (both visually and audible) congruent with their answer. An additional social evaluative threat is added by informing the participant that the researcher is standing in the room behind them and is monitoring their behaviour and (audibly) taking notes. The programme gives negative feedback to the subject “too slow” (visually) and “wrong” or “right” (both visually and audible). In addition the researcher gives feedback at 2 and 4 minutes (“Try to be a bit faster if possible” and “still one minute, try to earn as many points as possible” ). After the task, patients are instructed to RECOV for 15 minutes and the experimenter leaves the room. After the recovery phase, participants are instructed that the experiment will be repeated. They hence proceed to complete the identical control condition, stress and recovery phases for a second time.

Reference:

Spruyt, A., Clarysse, J., Vansteenwegen, D., Baeyens, F., & Hermans, D. (2009). Affect 4.0. Experimental psychology.

**Supplementary Information B**

Power Analysis

#for 60 people

> ##6. POWER ANALYSIS

> set.seed(123)

> #estimate the effect of Time

> model1

Linear mixed model fit by REML ['lmerMod']

Formula: y ~ Group + PSS + Time + (1 | Subject)

Data: covars

REML criterion at convergence: -618.7535

Random effects:

Groups Name Std.Dev.

Subject (Intercept) 0.3162

Residual 0.0500

Number of obs: 300, groups: Subject, 60

Fixed Effects:

(Intercept) GroupMDD PSS TimeRECOV1 TimeRECOV2 TimeSTR1 TimeSTR2

4.00 0.50 0.20 0.01 0.01 0.04 0.04

> sim_Time<- powerSim(model1, nsim=100, test = fixed("Time"))

> sim_Time

Power for predictor 'Time', (95% confidence interval):

100.0% (96.38, 100.0)

Test: Likelihood ratio

Based on 100 simulations, (0 warnings, 0 errors)

alpha = 0.05, nrow = 300

Time elapsed: 0 h 0 m 8 s

> #estimate power effect of Group

> model1

Linear mixed model fit by REML ['lmerMod']

Formula: y ~ Group + PSS + Time + (1 | Subject)

Data: covars

REML criterion at convergence: -618.7535

Random effects:

Groups Name Std.Dev.

Subject (Intercept) 0.3162

Residual 0.0500

Number of obs: 300, groups: Subject, 60

Fixed Effects:

(Intercept) GroupMDD PSS TimeRECOV1 TimeRECOV2 TimeSTR1 TimeSTR2

4.00 0.50 0.20 0.01 0.01 0.04 0.04

> sim_Group <- powerSim(model1, nsim=100, test = fixed("Group"))

> sim_Group

Power for predictor 'Group', (95% confidence interval):

100.0% (96.38, 100.0)

Test: Likelihood ratio

Based on 100 simulations, (0 warnings, 0 errors)

alpha = 0.05, nrow = 300

Time elapsed: 0 h 0 m 9 s

> #test a specific effect

> sim_interaction <- powerSim(model2, nsim=100, test = fixed("Group:Time"))

> sim_interaction

Power for predictor 'Group:Time', (95% confidence interval):

86.00% (77.63, 92.13)

Test: Kenward Roger (package pbkrtest)

Based on 100 simulations, (0 warnings, 0 errors)

alpha = 0.05, nrow = 300

Time elapsed: 0 h 0 m 14 s

**Supplementary Information C**

**Supplementary data comparing HR responses between baseline and control condition.** Since no significant difference occurred, and the baseline phase was more comparable to the recovery phases, the control condition was not regarded for computation of statistical comparisons. HC-Healthy Controls, MDD- Patients with MDD.

| **Differences between baseline and control conditions** | | | | |  |
| --- | --- | --- | --- | --- | --- |
| in HC | estimate | SE | Df | t.ratio | p value |
| BL - M1 | -0,012 | 0,015 | 172,000 | -0,778 | 0,437 |
| BL - M2 | -0,006 | 0,015 | 172,000 | -0,414 | 0,680 |
| in MDD |  |  |  |  |  |
| BL - M1 | -0,004 | 0,011 | 158,000 | -0,380 | 0,705 |
| BL - M2 | 0,007 | 0,011 | 158,000 | 0,599 | 0,550 |

**Supplementary Information D**

**Data on HF-HRV and LF-HRV**

In the following, data are presented on log(HF-HRV) and log(LF-HRV) (both as absolute power). Data for HF-HRV are highly comparable to the RMSSD in the main text.

| **phase** | |  | **HF-HRV** | | | **LF-HRV** | |
| --- | --- | --- | --- | --- | --- | --- | --- |
|  |  |  |  |  |  |  |  |
|  |  | *N* | *Group* | *mean* | *sd* | *mean* | *sd* |
| Run 1 | BL | 27 | HC | 553.8 | 569.28 | 807.87 | 592.64 |
|  |  | 26 | MDD | 416.08 | 567.33 | 765.67 | 754.65 |
|  | Stress 1 | 28 | HC | 493.6 | 424.54 | 716.98 | 494.93 |
|  |  | 26 | MDD | 410.48 | 483.36 | 701.6 | 526.57 |
|  | Recov 1 | 28 | HC | 648.22 | 556.29 | 1289.02 | 950.03 |
|  |  | 25 | MDD | 292.52 | 274.67 | 869.65 | 850.9 |
| Run 2 | Stress 2 | 28 | HC | 457.16 | 383.81 | 703.89 | 760.02 |
|  |  | 26 | MDD | 320.13 | 332.02 | 695.45 | 579.89 |
|  | Recov2 | 28 | HC | 672.94 | 459.51 | 1454.07 | 903.78 |
|  |  | 25 | MDD | 375.72 | 359.77 | 792.66 | 629.79 |

**ANOVA Table** on linear mixed model with log(**HF-HRV**) as dependent variable.

|  | **SumSq** | **Mean Sq** | **NumDF** | **DenDF** | **F** | **value** | **Pr(>F)** |
| --- | --- | --- | --- | --- | --- | --- | --- |
| group | 4.6199 | 4.6199 | 1 | 54.016 | 14.7806 | 0.00032 | *** |
| timepoint | 1.7805 | 0.4451 | 4 | 213.147 | 1.4242 | 0.227016 |  |
| PSS | 1.6941 | 1.6941 | 1 | 54.005 | 5.42 | 0.023682 | * |
| antidepressants | 0.013 | 0.013 | 1 | 54.188 | 0.0417 | 0.838928 |  |
| group:timepoint | 1.5354 | 0.3839 | 4 | 213.147 | 1.2281 | 0.299904 |  |
| group:PSS | 1.2724 | 1.2724 | 1 | 54.005 | 4.0708 | 0.048611 | * |

| **Post-hoc contrasts for log HF-HRV absolute power by group (emmeans).** Results are adjusted for PSS level. A trend occurred only in healthy controls: during the second stress task compared to the first recovery task and a significant increase during the second recovery task compared to the second stress task. No effect occurred in patients with MDD. P values are unadjusted. | | | | | | | |
| --- | --- | --- | --- | --- | --- | --- | --- |
| **Healthy Controls** | | | | | | | |
| contrast |  |  | estimate | SE | df | t.ratio | p.value |
| BL | - | S1 | 0.00851 | 0.154 | 222 | 0.055 | 0.956 |
| BL | - | R1 | -0.1553 | 0.154 | 222 | -1.008 | 0.3144 |
| BL | - | S2 | 0.14041 | 0.154 | 222 | 0.912 | 0.363 |
| BL | - | R2 | -0.26986 | 0.154 | 222 | -1.752 | 0.0812 |
| S1 | - | R1 | -0.16381 | 0.152 | 221 | -1.076 | 0.2833 |
| S1 | - | S2 | 0.1319 | 0.152 | 221 | 0.866 | 0.3874 |
| **R1** | **-** | **S2** | 0.29571 | 0.152 | 221 | 1.942 | **0.0535** |
| R1 | - | R2 | -0.11455 | 0.152 | 221 | -0.752 | 0.4528 |
| **S2** | **-** | **R2** | -0.41027 | 0.152 | 221 | -2.694 | **0.0076** |
|  |  |  |  |  |  |  |  |
| **Patients with MDD** | | | | | | | |
| contrast |  |  | estimate | SE | df | t.ratio | p.value |
| BL | - | S1 | -0.00947 | 0.158 | 221 | -0.06 | 0.9523 |
| BL | - | R1 | 0.06937 | 0.16 | 222 | 0.434 | 0.665 |
| BL | - | S2 | 0.17391 | 0.158 | 221 | 1.1 | 0.2724 |
| BL | - | R2 | 0.10351 | 0.16 | 221 | 0.647 | 0.5183 |
| S1 | - | R1 | 0.07884 | 0.16 | 222 | 0.493 | 0.6227 |
| S1 | - | S2 | 0.18338 | 0.158 | 221 | 1.16 | 0.2472 |
| R1 | - | S2 | 0.10455 | 0.16 | 222 | 0.653 | 0.5141 |
| R1 | - | R2 | 0.03414 | 0.162 | 222 | 0.211 | 0.8332 |
| S2 | - | R2 | -0.07041 | 0.16 | 221 | -0.44 | 0.6603 |

**ANOVA Table** on linear mixed model with log(**LF-HRV**) as dependent variable.

|  | SumSq | Mean Sq | NumDF | DenDF | F | value | Pr(>F) |
| --- | --- | --- | --- | --- | --- | --- | --- |
| group | 2151640 | 2151640 | 1 | 53.811 | 7.5118 | 0.008301 | ** |
| timepoint | 8576553 | 2144138 | 4 | 213.215 | 7.4856 | 1.16E-05 | *** |
| PSS | 763919 | 763919 | 1 | 53.777 | 2.667 | 0.108291 |  |
| antidepressants | 29473 | 29473 | 1 | 54.329 | 0.1029 | 0.74961 |  |
| group:timepoint | 4862256 | 1215564 | 4 | 213.215 | 4.2438 | 0.002516 | ** |
| group:PSS | 723069 | 723069 | 1 | 53.777 | 2.5244 | 0.117961 |  |

**Post-hoc contrasts for log LF-HRV absolute power by group (emmeans).**

| **Healthy Controls** | |  |  |  |  |  |  |
| --- | --- | --- | --- | --- | --- | --- | --- |
| contrast |  |  | estimate | SE | df | t.ratio | p.value |
| BL | - | S1 | 112.09 | 147 | 222 | 0.76 | 0.4478 |
| BL | - | R1 | -459.96 | 147 | 222 | -3.121 | 0.002 |
| BL | - | S2 | 125.18 | 147 | 222 | 0.849 | 0.3966 |
| **BL** | **-** | **R2** | -625.01 | 147 | 222 | -4.24 | **<.0001** |
| **S1** | **-** | **R1** | -572.04 | 146 | 221 | -3.924 | **0.0001** |
| S1 | - | S2 | 13.09 | 146 | 221 | 0.09 | 0.9286 |
| **R1** | **-** | **S2** | 585.13 | 146 | 221 | 4.013 | **0.0001** |
| R1 | - | R2 | -165.06 | 146 | 221 | -1.132 | 0.2588 |
| **S2** | **-** | **R2** | -750.19 | 146 | 221 | -5.145 | **<.0001** |
|  |  |  |  |  |  |  |  |
| **Patients with MDD** | |  |  |  |  |  |  |
| contrast |  |  | estimate | SE | df | t.ratio | p.value |
| BL | - | S1 | 64.07 | 151 | 221 | 0.423 | 0.6724 |
| BL | - | R1 | -103.5 | 153 | 222 | -0.676 | 0.4997 |
| BL | - | S2 | 70.22 | 151 | 221 | 0.464 | 0.643 |
| BL | - | R2 | -1.97 | 153 | 222 | -0.013 | 0.9897 |
| S1 | - | R1 | -167.57 | 153 | 222 | -1.095 | 0.2749 |
| S1 | - | S2 | 6.15 | 151 | 221 | 0.041 | 0.9676 |
| R1 | - | S2 | 173.72 | 153 | 222 | 1.135 | 0.2577 |
| R1 | - | R2 | 101.52 | 155 | 222 | 0.655 | 0.5129 |
| S2 | - | R2 | -72.2 | 153 | 222 | -0.472 | 0.6377 |

*Note: 4 participants (all MDD patients) were identified who had a breathing frequency outside of the 9 to 24 breaths/minute range in at least 1 phase and were eliminated in a secondary analysis. Results are reported below.

For patients with MDD log(HF-HRV)(inside breathing frequency):

Type III Analysis of Variance Table with Satterthwaite's method

Sum Sq Mean Sq NumDF DenDF F value Pr(>F)

timepoint 1.42913 0.35728 4 86.100 1.1564 0.33579

PSS 0.56929 0.56929 1 22.110 1.8426 0.18834

HAMD 0.00086 0.00086 1 22.005 0.0028 0.95852

antidepr. 0.15008 0.15008 1 22.110 0.4858 0.49309

Activity 0.00040 0.00040 1 22.116 0.0013 0.97173

Smoke 1.01962 1.01962 1 22.007 3.3002 0.08292

Contracept 0.08028 0.08028 1 22.098 0.2598 0.61528

---

Signif. codes: 0 ‘***’ 0.001 ‘**’ 0.01 ‘*’ 0.05 ‘.’ 0.1 ‘ ’ 1

**Supplementary Figure 1**


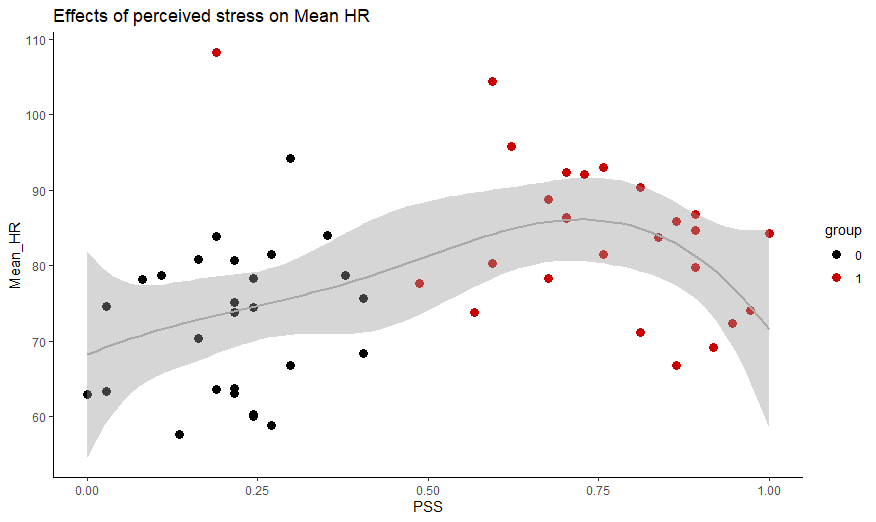


**Supplementary Table 1.** **Contrasts Subjective Stress.** Contrasts were derived from linear mixed models separately for each group. Within each group, p values are adjusted for multiple testing using Holms's correction. BL=Baseline, RECOV=recovery phase. In healthy controls stress levels rose by a mean of 2.92 points from baseline to the first stressor, in patients stress ratings rose around 2.44 points.

|  | **Controls** | | | | | |  |  |  | **MDD** |  |  |  |  |
| --- | --- | --- | --- | --- | --- | --- | --- | --- | --- | --- | --- | --- | --- | --- |
| **Contrast** | **estimate** | **SE** | **df** | **t ratio** | **p** | **q** |  | **estimate** | **SE** | **df** | **t ratio** | **p** | **p.adj** |  |
| BL - RELAX1 | -0,57 | 0,39 | 228,30 | -1,48 | 0,141 | 0,154 |  | -1,56 | 0,39 | 228,30 | -3,95 | 0,00 | 0,000 |  |
| BL - RELAX2 | -0,14 | 0,39 | 228,30 | -0,37 | 0,712 | 0,712 |  | -1,19 | 0,39 | 228,30 | -3,01 | 0,00 | 0,004 |  |
| BL - STRESS1 | -2,93 | 0,39 | 228,30 | -7,57 | 0,000 | 0,000 |  | -2,44 | 0,39 | 228,30 | -6,21 | 0,00 | 0,000 |  |
| BL - STRESS2 | -2,57 | 0,39 | 228,30 | -6,65 | 0,000 | 0,000 |  | -2,56 | 0,39 | 228,30 | -6,49 | 0,00 | 0,000 |  |
| RELAX1- STRESS1 | -2,36 | 0,39 | 228,30 | -6,10 | 0,000 | 0,000 |  | -0,89 | 0,39 | 228,30 | -2,26 | 0,02 | 0,030 |  |
| RELAX2- STRESS2 | -2,43 | 0,39 | 228,30 | -6,28 | 0,000 | 0,000 |  | -1,37 | 0,39 | 228,30 | -3,48 | 0,00 | 0,001 |  |

| **Supplementary Table 2** |  |  |  |  |  |  |  |  |  |
| --- | --- | --- | --- | --- | --- | --- | --- | --- | --- |
| Mean levels of HR and RMSSD per group and phase | | | | | | | | | |
|  | Phase |  |  | HR | | RMSSD | | HR MDD vs. HC | RMSSD MDD vs. HC |
|  |  |  |  |  |  |  |  |  |  |
|  |  | Group | n | mean | Sd | mean | sd | p | **p** |
| Run 1 | BL | HC | 27 | 72,28 | 9,42 | 34,11 | 13,76 | **<0,001** | **0,018** |
|  |  | MDD | 26 | 84,14 | 10,16 | 25,53 | 14,32 | - | **-** |
|  | Stress 1 | HC | 28 | 77,64 | 9,83 | 38,53 | 15,06 | **<0,001** | **<0,001** |
|  |  | MDD | 26 | 88,13 | 11,98 | 24,05 | 12,10 | - | **-** |
|  | Recov 1 | HC | 28 | 70,92 | 8,59 | 37,04 | 14,49 | **<0.001** | **<0.001** |
| Run 2 |  | MDD | 25 | 84,09 | 9,43 | 23,91 | 11,55 | **-** | **-** |
|  | Stress 2 | HC | 28 | 76,29 | 8,97 | 30,59 | 11,25 | **0,002** | **0,022** |
|  |  | MDD | 26 | 85,08 | 10,83 | 23,8 | 12,51 | **-** | **-** |
|  | Recov2 | HC | 28 | 69,67 | 8,04 | 40,24 | 14,97 | **<0.001** | **0,002** |
|  |  | MDD | 25 | 82,44 | 10,64 | 26,76 | 12,75 | - | **-** |
| Note: Values represent averaged HR/RMSSD by groups. Mean values reflect data of all available subjects per phase. Welch’s T-test was performed on log transformed variables. Throughout the paradigm, the baseline group differences | | | | | | | | | |

| **Supplementary table 3.** Results of confusion matrix for classification of individuals based on HR, RMSSD or HR and RMSSD combined. | | | | |
| --- | --- | --- | --- | --- |
| **Phase** | **Sensitivity** | **Specificity** | **Classification Accuracy** | **p-value (accuracy>no information rate)** |
| **Heart Rate** | | | | |
| **Baseline** | 62,96 | 69,23 | 66,04 | **0.019*** |
| **Stress 1** | 67,86 | 65,38 | 66,67 | **0.020*** |
| **Stress 2** | 71,43 | 65,38 | 68,52 | **0.010*** |
| **Recovery 1** | 78,57 | 72 | 75,47 | **<0.001***** |
| **Recovery 2** | 75 | 72 | 73,58 | **0.002**** |
| **RMSSD** | | | | |
| **Baseline** | 66,67 | 53,85 | 60,38 | 0,107 |
| **Stress 1** | 78,57 | 56 | 67,92 | **0.019*** |
| **Stress 2** | 71,43 | 50 | 61,11 | 0,11 |
| **Recovery 1** | 78,57 | 56 | 67,92 | **0.019*** |
| **Recovery 2** | 78,57 | 48 | 64,15 | 0,064 |
| **Combined RMSSD+HR** | | | | |
| **Baseline** | 62,96 | 73,07 | 67,92 | **0.009*** |
| **Stress 1** | 68 | 71,43 | 69,81 | **0.009*** |
| **Stress 2** | 71,43 | 64,38 | 68,52 | **0.010*** |
| **Recovery 1** | 82,14 | 72 | 77,36 | **<0.001***** |
| **Recovery 2** | 82,14 | 68 | 75,47 | **0.001**** |
|  |  |  |  |  |
